# Quackery in Turkey

**Published:** 1865-07

**Authors:** 


QUACKERY IN TURKEY.—In consequence of the death of a soldier having occurred in Constantinople, after taking the prescription of a quack doctor, the medical director-general ordered a commission of inquiry into the quality of the medicine. The result has been a seizure of the whole of his drugs, he himself being arrested for illegal practice.

